# Crosslinked Polyarylene Ether Nitrile Interpenetrating with Zinc Ion Bridged Graphene Sheet and Carbon Nanotube Network

**DOI:** 10.3390/polym9080342

**Published:** 2017-08-04

**Authors:** Renbo Wei, Jialing Wang, Hongxing Zhang, Weihua Han, Xiaobo Liu

**Affiliations:** Research Branch of Advanced Functional Materials, School of Microelectronics and Solid-State Electronics, High Temperature Resistant Polymer and Composites Key Laboratory of Sichuan Province, University of Electronic Science and Technology of China, Chengdu 610054, China; 18702893579@163.com (J.W.); zhx78668@126.com (H.Z.); hwh450@sohu.com (W.H.)

**Keywords:** interpenetrating network, crosslinked polyarylene ether nitrile, graphene sheet, carbon nanotube, dielectric properties

## Abstract

We report the fabrication and improved properties of crosslinked polyarylene ether nitrile (CPEN) interpenetrating with a zinc ion bridged graphene sheet (GS) and carbon nanotube (CNT) network (GS-Zn-CNT) (CPEN/GS-Zn-CNT). Graphene oxide (GO) and acidulated CNT were firstly prepared and then coordinated with zinc ions to form the zinc ion bridged GO and CNT network (GO-Zn-CNT). The mass ratio of GO and acidulated CNT in GO-Zn-CNT was controlled to be 1:3 and the optimized content of Zn^2+^ was Zn^2+^/C = 0.01 mmol/mg (mole of zinc acetate/total weight of GO and acidulated CNT). Phthalonitrile end-capped polyarylene ether nitrile (PEN-Ph) permeated into the GO-Zn-CNT in *N*-methyl-2-pyrrolidone (NMP) and the corresponding composite PEN/GO-Zn-CNT was fabricated through the solution-casting method. After thermal annealing at 230 °C for 1 h and further curing at 320 °C for 2 h, the GO in GO-Zn-CNT was partly reduced into GS, and PEN-Ph was crosslinked, offering the CPEN/GS-Zn-CNT. The mechanical, thermal, and electrical properties of the obtained CPEN/GS-Zn-CNT were investigated in detail. The glass transition temperature, relative permittivity, and tensile strength of CPEN/GS-Zn-CNT with 2.0 wt % GS-Zn-CNT, compared to that of PEN, were increased by 18%, 181%, and 27%, respectively. The CPEN/GS-Zn-CNT based composite is a potential candidate as material in high performance electronic devices.

## 1. Introduction

Polyarylene ether nitrile (PEN) has attracted extensive attention from the scientific research community owing to its outstanding properties, including radiation resistance, good mechanical properties, and high thermal stability [1,2,3]. PEN has shown potential applications in the areas of elevated temperature and aggressive chemical environments. However, further application in special areas including aerospace, military, and other extreme conditions is still a challenge without the modification of the PEN. For example, pure PEN cannot be used at temperatures higher than 250 °C because its intrinsic glass transition temperature is lower than that temperature [4,5,6,7]. What is more, although the extensive existing of -CN on the side-chain of PEN can promote the dielectric properties of PEN effectively, the relative permittivity (~3–4) of PEN is still limited to application in electrical equipment. As a result, all kinds of techniques, such as chemical grafting, surface modification, physical blending, as well as crosslinking, have been adopted to improve the physical performance of PEN so that it can be used in these conditions [3,4,5].

With the advantage of straight forward preparation, blending of PEN with other additivse is widely used. Up to now, additives including polyaniline [8], copper phthalocyanine [9], barium titanate [10,11], titanium dioxide [12], carbon nanotubes CNTs [13], graphene/graphene oxide [14], among others, have been incorporated into the PEN matrix to obtain the corresponding composites with improved physical properties. Especially, the carbon materials (CNT and graphene) have attracted extensive attention in the PEN based composites resulting from their outstanding thermal, mechanical, electrical, and optical properties. To avoid the aggregation of CNT and graphene, which results from the Van der Walls and π–π interactions between them at high concentrations in the PEN matrix, three dimensional CNT-graphene networks have been constructed [15]. In addition to acting as the traditional polymeric additives, the constructed CNT-graphene networks can be permeated by PEN chains, resulting in semi-interpenetrating networks. The thermal and dielectric properties of the obtained semi-interpenetrating networks have been effectively improved compared with those of pristine PEN.

Crosslinking is another generally used method to improve the physical properties of linear polymers. After the crosslinking reaction, the linear or mildly branched macromolecules convert into a three-dimensional network structure and the strength, heat resistance, abrasion resistance, solvent resistance, and other properties of the obtained system are effectively improved [16]. To crosslink the polyarylene ether nitrile, polyarylene ether nitrile terminated with phthalonitrile (PEN-Ph) is designed and synthesized [17]. Through simply curing at high temperature and/or catalyzing with (metal) ions (Cu^2+^, Zn^2+^, Fe^3+^, and others), the phthalonitrile is capped at the ends of PEN self-crosslinks, forming the metal phthalocyanines as the crosslinking points in the system. Although the obtained network is a PEN elastomer as the crosslinking points are at the ends of linear PEN, the glass transition temperature of the obtained system increases by more than 100 °C [3].

Interpenetrating polymer networks (IPNs) are elaborated as a special class of polymers which combine two polymers in which one is synthesized or polymerized in the presence of the other [18,19,20,21]. Different from physical blends and copolymerization, IPNs are a new way to combine two different polymers which could be non-compatible polymers with different properties, and/or polymers with opposite properties like hydrophilicity/hydrophobicity. Fabrication of IPNs is a useful method to develop new products with excellent physical and mechanical properties, more so than the normal polymeric blends [20]. By combining the three dimensional CNT-graphene network as the additive and the PEN-Ph permeates into this network followed by crosslinking, an organic-inorganic interpenetrating network could be imagined.

In this paper, we report the fabrication of a crosslinked polyarylene ether nitrile (CPEN) interpenetrating with a zinc ion bridged graphene sheet (GS) and carbon nanotube network (CPEN/GS-Zn-CNT). Zinc ion bridged graphene oxide (GO) and CNT network (GO-Zn-CNT) are firstly prepared from GO and acidulated CNT. Phthalonitrile end-capped polyarylene ether nitrile permeates into the GO-Zn-CNT in *N*-methyl-2-pyrrolidone (NMP) and the corresponding composite PEN/GO-Zn-CNT is fabricated through the solution-casting method. After the thermal annealing at 230 °C and further curing at 320 °C, the CPEN/GS-Zn-CNT is successfully fabricated. The fabrication and improved properties of the obtained CPEN/GS-Zn-CNT are investigated in detail.

## 2. Materials and Methods

### 2.1. Materials

Purified multi-walled CNT (MWCNT) (outer diameter: about 50 nm; length: 30–50 μm; purity: 95 wt %) was purchased from Chengdu Organic Chemistry, Chinese Academy of Sciences, Chengdu, China. GO was purchased from Jiangsu Chaodian Co. Ltd., Jiangsu, China. Zinc acetate (99%) was purchased from Kelong reagent Co. Ltd., Chengdu, China. *N*-methyl-2-pyrrolidone (NMP) was purchased from Chengdu KeLong chemicals, Chengdu, China. All other reagents were commercially available products and used as received without further purification.

### 2.2. Preparation of PEN-Ph

**Figure 1:**
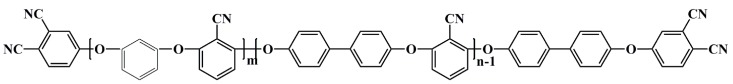
The structure of the phthalonitrile end-capped polyarylene ether nitrile (PEN-Ph).

The structure of phthalonitrile end-capped polyarylene ether nitrile (PEN-Ph) is shown in Figure 1. PEN-Ph was synthesized in our laboratory by a nucleophilic substitution reaction with biphenyl (BP), hydroquinone (HQ) and 2,6-dichlorobenzonitrile (DCBN), followed by termination with 4-nitrophthalonitrile [9]. It was purified several times by precipitating from ethanol to remove the unreacted monomers before use. The number average molecular weight of the obtained PEN-Ph is 3.4 × 10^4^ and the PDI is 1.46. The purity of the PEN-Ph is 100% according to the ^1^H NMR results.

### 2.3. Preparation of the CPEN/GS-Zn-CNT

GO (25 mg) and acidulated MWCNT (75 mg) were added into 30 mL NMP solution of PEN-Ph (2.0 g), and the total mass of GO and acidulated MWCNT in the composite PEN/GO-Zn-CNT was 0.0, 0.5, 1.0, 1.5, and 2.0 wt %, respectively. Simultaneously, the mixture was added into a three-neck flask, and then the flask was put into the tank (full of water) of the sonication machine (40 kHz, 100 W) for sonication for 2 h to get a homogeneous dispersion. Then, zinc acetate was added into the above-mentioned mixture with mechanical stirring for 24 h, and then with high-intensity sonication (40 kHz, 200 W) for another 1 h. After that, the mixture was cast on a clean glass plate, and dried in an oven at 200 °C to obtain the composite films. Moreover, the films were thermally annealed at 230 °C for 1 h to obtain PEN/GS-Zn-CNT composite films. Finally, the PEN/GS-Zn-CNT composite films were further cured at 320 °C for 2 h to obtain the interpenetrating network CPEN/GS-Zn-CNT.

As a control experiment, the inorganic network GO-Zn-CNT without PEN-Ph was also fabricated. Firstly, GO (25 mg) and acidulated MWCNT (75 mg) were added into 30 mL NMP solution. To investigate the coordination of zinc ion with GO and CNT, zinc acetate was added into the dispersion, the Zn^2+^/C was controlled to be 0.001, 0.002, 0.005, 0.01, 0.02 and 0.04 mmol/mg. The mixture was left for coordination for 24 h. After that, the coordination system was immersed in a liquid nitrogen bath to freeze for 3 min. After being fully frozen, the sample was settled in the sealed thermal baffles of expanded polystyrene with an ultralow vacuum chamber (−55 °C, <10 Pa, LGJ-10 Freeze Drier, 970 W, Beijing Huaxing Technology Development Co., Ltd., Songyuan, China). The final 3D GO-Zn-CNT was obtained by a subsequent drying process for 48 h.

### 2.4. Characterization

The micro-morphologies of the CPEN/GS-Zn-CNT composite films were characterized by scanning electron microscope (SEM, FEI INSPECT F50, Hillsboro, OR, USA). For fracturing, the film was clamped in liquid nitrogen inside the vacuum cup, fracturing was achieved by folding the film. The samples prepared for SEM studies were observed after sputter coating treatment with Au. The crystal structure of CPEN/GS-Zn-CNT composite films were characterized by X-ray film diffraction (XRD, Rigaku RINI2400 with Cu Kα radiation). Differential scanning calorimetry (DSC) analysis was performed on a DSC system of a TA instrument (Q100, TA Instruments Ltd., New Castle, DE, USA) under nitrogen atmosphere at a heating rate of 10 °C/min. Thermogravimetric analysis (TGA) of the PEN based composite films was performed at a heating rate of 20 °C/min from room temperature to 800 °C, using a TA instrument Q50 series analyzer system combined with a data processing station (TA Instruments Ltd., New Castle, DE, USA). Dielectric properties for the PEN based composite films were monitored according to the ASTM D150 on a TH 2819A precision Lenze Capacitor Resistance (LCR) meter (Tonghui Electronic Co., Ltd., Dongguan, China). Mechanical properties of the samples were tested with a SANS CMT6104 series desktop electromechanical universal testing machine (Shenzhen Sans Materials Testing Machine Co., Shenzhen, China), the films were cut into standard strips (10 mm × 100 mm), and the stretching speed was 5 mm/min at room temperature, the results were gained as an average value for every five samples.

## 3. Results and Discussion

In this study, the fabrication and the improved properties of the organic-inorganic interpenetrating network CPEN/GS-Zn-CNT, crosslinked polyarylene ether nitrile as the organic network and the zinc ion bridged graphene sheet and carbon nanotube network as the inorganic network, are investigated in detail. In order to study the influence of the GS-Zn-CNT on the thermal, mechanical, and dielectric properties of the CPEN/GS-Zn-CNT, four CPEN/GS-Zn-CNT composites with different content of GS-Zn-CNT were fabricated. In addition, the crosslinked polyarylene ether nitrile (CPEN) and the zinc ion bridged graphene sheet and carbon nanotube network (GS-Zn-CNT) were also prepared for comparison. The CPEN/GS-Zn-CNT and the related intermediates were characterized by SEM observation, photograph comparison, thermal measurement, mechanical testing, and dielectric analysis. The analytical results are discussed below.

### 3.1. Fabrication and Characterization of GO-Zn-CNT

**Figure 2:**
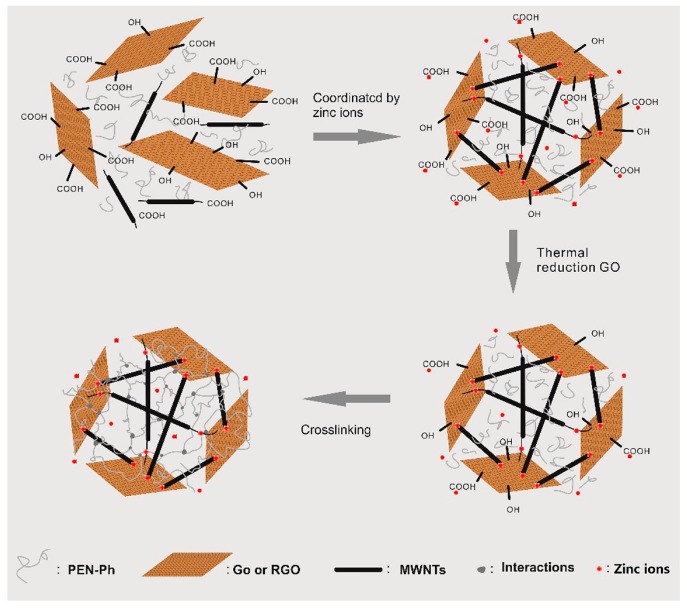
Fabrication route of the CPEN/GS-Zn-CNT. CPEN = crosslinked polyarylene ether nitrile; GS = graphene sheet; Zn = zinc; CNT = carbon nanotube.

The fabrication route of the CPEN/GS-Zn-CNT is illustrated as Figure 2. Firstly, GO and acidulated CNT were obtained and dispersed in the NMP solution of PEN-Ph. After the addition of zinc acetate, the zinc ions coordinate with the oxygen containing groups (hydroxyl group, epoxy group, and carboxyl group) forming the inorganic network (GO-Zn-CNT). A similar result was reported by Tong et al. [15], who obtained GO and CNT network by coordinating with copper ions. Liu et al. [22] also reported the fabrication of carbon nanotube/graphene networks coordinated by divalent metal (Cu, Ca or Mg) ions. On the other hand, PEN-Ph permeates into the GO-Zn-CNT as the GO and acidulated CNT are dispersed in the NMP solution of PEN-Ph. Through the solution-casting method, the composite PEN/GO-Zn-CNT was obtained. With the thermal annealing at 230 °C for 1 h, the GO in the composite was partly reduced into GS, forming PEN/GS-Zn-CNT. Finally, the CPEN/GS-Zn-CNT was obtained by further curing at 320 °C for 2 h through which the PEN-Ph self-crosslinks and/or is catalyzed by zinc ions with the presence of GS-Zn-CNT. The crosslinking of PEN-Ph at high temperature and/or catalyzing by metal ions has been reported elsewhere [23,24].

**Figure 3:**
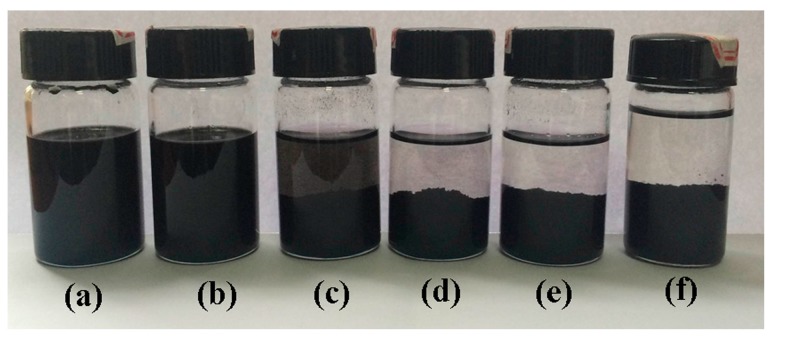
The photograph of CNT and graphene oxide (GO) dispersions with different amount of Zn^2+^; the ratios of Zn^2+^ (mmol)/C (mg) for (**a**), (**b**), (**c**), (**d**), (**e**), and (**f**) are 0.001, 0.002, 0.005, 0.01, 0.02, and 0.04, respectively.

The inorganic network GO-Zn-CNT was firstly studied without PEN-Ph. The mass ratio of GO and acidulated CNT was 1:3 due to the fact that at this ratio the copper ion bridge reduced GO and CNT network (GS-Cu-CNT) shows the best electrical properties [22]. To investigate the coordination of zinc ion with GO and CNT, zinc acetate was added into a 30 mL dispersion of GO (25 mg) and acidulated CNT (75 mg), the Zn^2+^/C is controlled to be 0.001, 0.002, 0.005, 0.01, 0.02, and 0.04 mmol/mg. Figure 3 shows the photographs of GO-Zn-CNT after coordination for 24 h. When the Zn^2+^/C is higher than 0.01 mmol/mg, the corresponding hybrid system precipitates completely, meaning the CNT and GO are bridged by the Zn^2+^ forming GO-Zn-CNT networks. At Zn^2+^/C = 0.005 mmol/mg, some of the GO-Zn-CNT precipitates and then precipitates completely after 48 h. When the Zn^2+^/C is less than 0.002 mmol/mg, however, the GO-Zn-CNT can be uniform and stable for several days. Usually, at a higher concentration, zinc ions are easier to coordinate with GO and acidulated CNT to form the GO-Zn-CNT, which translated into a shorter precipitation time. As a result, Zn^2+^/C should be higher than 0.005 mmol/mg to make sure that all of the GO and CNT can be coordinated during the sample fabrication procedure.

**Figure 4:**
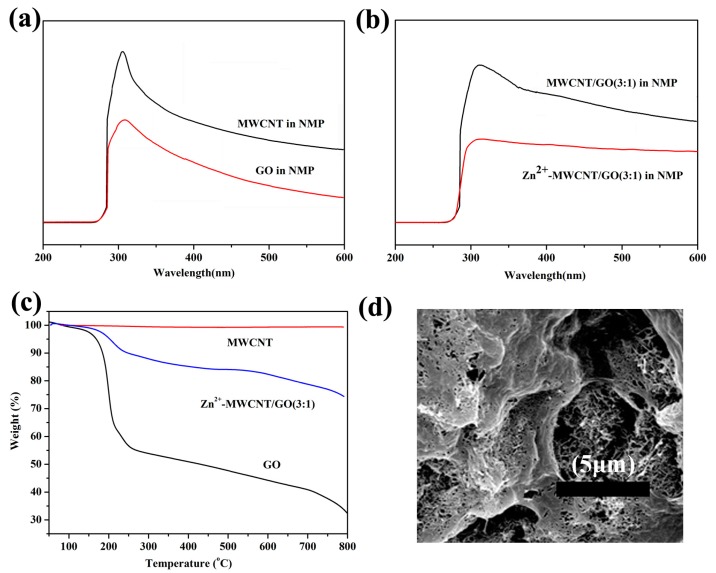
UV-vis spectra of (**a**) multi-walled CNTs (MWCNTs) and GO dissolved in *N*-methyl-2-pyrrolidone (NMP), (**b**) MWCNT/GO (3/1) and Zn^2+^-coordinated MWCNT/GO (3/1) dissolved in NMP, (**c**) the TGA curve of the MWCNT, Zn^2+^-coordinated MWCNT/GO (3/1) and GO, (**d**) the SEM micrographs of the Zn^2+^-coordinated MWCNT/GO (3/1).

The GO-Zn-CNT is characterized by UV-vis. As shown in Figure 4a, both GO and acidulated MWCNT show a peak at around 302 nm which corresponds to the n–π* transition of the C=O bonds, indicating that these carbon materials are well dispersed in NMP. The UV-vis spectrum is almost the same when GO and acidulated CNT are mixed together at the mass ratio of 1:3 (Figure 4b). After the addition of zinc acetate, the strength of the peak around 302 nm decreases, indicating the consumption of the C=O bonds which are coordinated with the zinc ions. Ruoff et al. [25] reported the reduction of the C=O bonds on GO through coordination with Ca^2+^ and Mg^2+^. TGA was also incorporated to characterize the coordination between Zn^2+^ and GO as well as CNT (Figure 4c). CNT shows excellent thermal stability as no obvious weight loss was observed on its TGA curve. In comparison, GO suffers a significant weight loss in the temperature range from 160 to 250 °C, attributable to the decomposition of labile oxygen functional groups on the basal plane. After the coordination with zinc ions, the weight loss of the GO-Zn-CNT shifts to a higher temperature range, resulting from the coordination effect. In addition, the network of GO-Zn-CNT was also confirmed by SEM observation after the freeze-drying procedure. As shown in Figure 4d, the CNTs interweave between different GO sheets, forming three-dimensional network structures. Similar results were also reported by Tong et al. [15] and Liu et al. [22] by using copper ions instead of zinc ions.

### 3.2. Fabrication and Characterization of CPEN/GS-Zn-CNT

**Figure 5:**
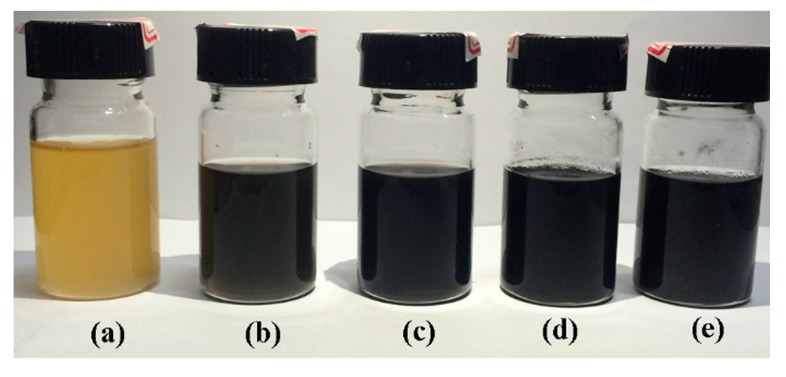
The photograph of the GO-Zn-CNT in NMP solution of PEN-Ph. The Zn^2+^/C is controlled to be 0.01 mmol/mg, the mass ratio of GO and CNT to PEN (C/PEN) is 0/100 (**a**); 0.5/99.5 (**b**); 1/99 (**c**); 1.5/98.5 (**d**); and 2/98 (**e**).

After the investigation of the inorganic network GO-Zn-CNT, the organic part was incorporated. The crosslinkable polyarylene ether nitrile (PEN-Ph) was dissolved in the NMP solvent, which was used to disperse the GO and acidulated CNT to make sure the PEN-Ph could permeate thoroughly into GO-Zn-CNT. Figure 5 shows the photograph of GO-Zn-CNT in NMP solution of PEN-Ph. The Zn^2+^/C was controlled to be 0.01 mmol/mg so that all the GO and CNT could be coordinated and the subsequent hybrid system could be uniform and stable during the sample fabrication procedure. The mass ratio of GO and CNT to PEN (C/PEN) was 0/100, 0.5/99.5, 1/99, 1.5/98.5, and 2/98. In comparison with the sample in Figure 3d, all of these zinc ion coordinated systems were uniform and stable for 2–3 days due to the existence of PEN-Ph. Such a long stable time is sufficient for the subsequent solution-casting procedure.

**Figure 6:**
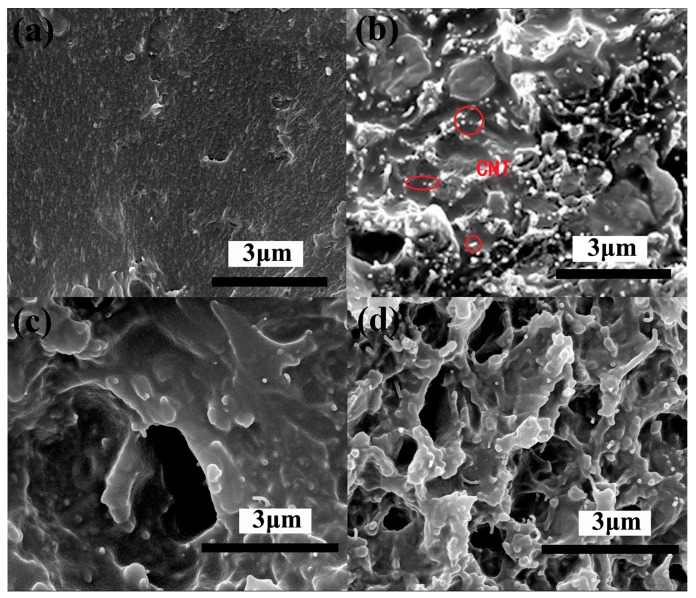
SEM micrographs of cross-sectional morphologies of (**a**) PEN; (**b**) PEN/GO-Zn-CNT20; (**c**) PEN/GS-Zn-CNT20; (**d**) CPEN/GS-Zn-CNT20.

The PEN-Ph and GO-Zn-CNT based composites (PEN/GO-Zn-CNT0, PEN/GO-Zn-CNT5, PEN/GO-Zn-CNT10, PEN/GO-Zn-CNT15, and PEN/GO-Zn-CNT20 with 0, 0.5, 1.0, 1.5, and 2.0 wt % of GO and CNT, respectively) were obtained by a solution-casting method as reported in the literature [15]. The morphologies of the obtained PEN/GO-Zn-CNT were characterized by SEM observation. Figure 6 shows the typical SEM micrographs of morphologies of PEN and PEN/GO-Zn-CNT20. The morphology of PEN was relatively smooth, representing a typical one-component phase structure (Figure 6a). In comparison, a rough ductile fracture morphology was observed for PEN/GO-Zn-CNT20 (Figure 6b). Although the GO nanosheets cannot be identified from the PEN matrix, the CNTs were homogeneously dispersed in the PEN, indicating the uniform dispersion of the GO and CNT network in PEN. In addition, the inner space of the GO-Zn-CNT was filled with the continuous PEN matrix, forming the semi-interpenetrating network PEN/GO-Zn-CNT.

### 3.3. Dielectric Properties of the Composites

**Figure 7:**
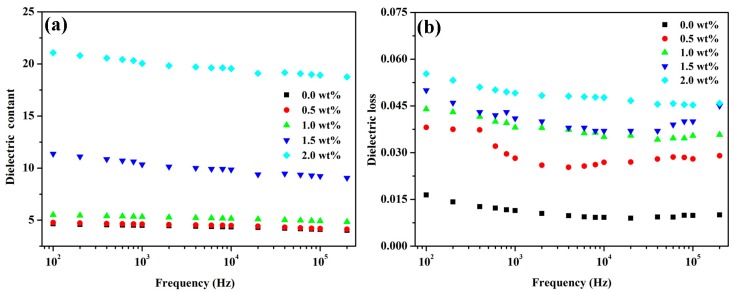
The relative permittivity (**a**) and dielectric loss (**b**) of the PEN/GO-Zn-CNT based composites.

The relative permittivity and dielectric loss of the pure PEN and PEN/GO-Zn-CNT composite films were measured at room temperature as a function of frequency (Figure 7). For PEN, the relative permittivity was almost the same (~4.0) in the measured frequency range of 250 Hz–200 kHz. The relative permittivity of the composite films decreased slightly with increasing measuring frequency. This phenomenon was caused by the effect of the polarization relaxation which has been reported elsewhere [26,27,28]. For the composites PEN/GO-Zn-CNT, the relative permittivity of the composites increases enormously compared with that of PEN, this was due to the electric conductivities of CNT and GO. Specifically, the relative permittivity of the composite PEN/GO-Zn-CNT20 reached 22 (100 Hz), with an increment of 450% in comparison with that of PEN, as shown in Figure 7a. Similar results were also observed for the dielectric loss of the PEN/GO-Zn-CNT composite films (Figure 7b). Although the dielectric loss increased with increasing content of GO and CNT, all of the PEN/GO-Zn-CNT composite films showed relatively low dielectric loss (<0.06), which was only 0.05 for PEN/GO-Zn-CNT20 at 1 kHz.

**Figure 8:**
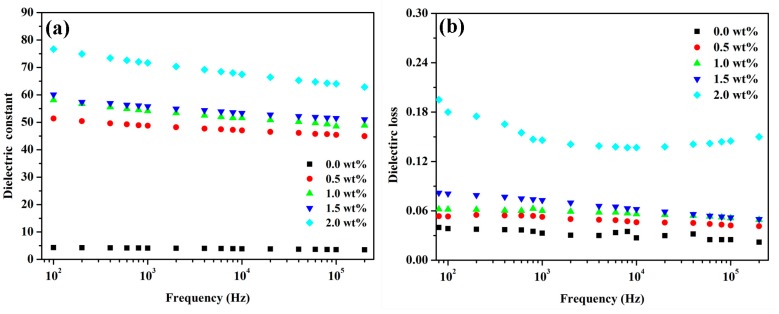
The relative permittivity (**a**) and dielectric loss (**b**) of the PEN based composites annealed at 230 °C for 1 h.

Compared to pure graphene or reduced graphene oxide, the hydrophilic oxygen groups (epoxide, hydroxy, and carboxylic groups) on the basal plane and edge of GO can effectively promote its dispersion and adhesion in/to the polymer matrixes. However, GO shows low electrical conductivity which limits its use for the preparation of conductive or dielectric nanocomposites. Fortunately, the electrical conductivity of graphene oxide can be significantly increased by chemical reduction and thermal reduction. To improve the dielectric properties of the PEN based composites, the PEN/GO-Zn-CNT were thermally annealed at 230 °C for 1 h through which the GO in GO-Zn-CNT was partly reduced into GS, offering PEN/GS-Zn-CNT. The relative permittivity and dielectric loss of the PEN/GS-Zn-CNT composite films are shown in Figure 8. Compared to PEN/GO-Zn-CNT, the relative permittivity of PEN/GS-Zn-CNT increased (Figure 8a). Specifically, the relative permittivity of the composite PEN/GS-Zn-CNT20 reached 78 (100 Hz), with an increment of 250% compared with that of PEN/GO-Zn-CNT20. The dielectric loss of PEN/GS-Zn-CNT also increased after the thermal annealing (Figure 8b). However, it was much lower than the PEN/graphene composite at the same content of additives reported in the literature [29].

**Figure 9:**
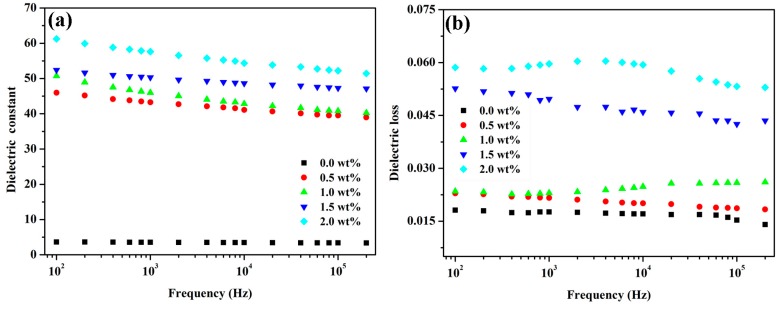
The relative permittivity (**a**) and dielectric loss (**b**) of the PEN based composites cured at 320 °C for 2 h.

The phthalonitrile groups capped at the ends of PEN-Ph can be crosslinked, forming phthalocyanines as the crosslinking points in the system, through curing at high temperature and/or catalyzing with (metal) ions (Cu^2+^, Zn^2+^, Fe^3+^, and others) [30,31,32]. As a result, to further improve the dielectric properties of the PEN and GS-Zn-CNT based composites, they were cured at 320 °C for 2 h to obtain the interpenetrating networks CPEN/GS-Zn-CNT. The crosslinking of the PEN-Ph has been reported by Yang et al. [3] and can be confirmed by dissolving the CPEN/GS-Zn-CNT in NMP. The result showed that the CPEN/GS-Zn-CNT cannot be dissolved by NMP even for three days where as the PEN/GS-Zn-CNT can be quickly dissolved in 3–8 h. Figure 9 shows the relative permittivity and dielectric loss of the CPEN/GS-Zn-CNT composite films. Compared to the PEN/GS-Zn-CNT, the relative permittivity decreases after the curing treatment (Figure 9a). Specifically, the relative permittivity of the composite CPEN/GS-Zn-CNT20 decreased to 62 (100 Hz), but was still 14.5 times larger than that of pure PEN. The decrease of the relative permittivity is a result of the crosslinking of the PEN-Ph which constrains the polarization of the polar groups in the obtained system. The dielectric loss of CPEN/GS-Zn-CNT also decreased after the curing treatment (Figure 9b).

### 3.4. Thermal and Mechanical Properties of the Composites

**Figure 10:**
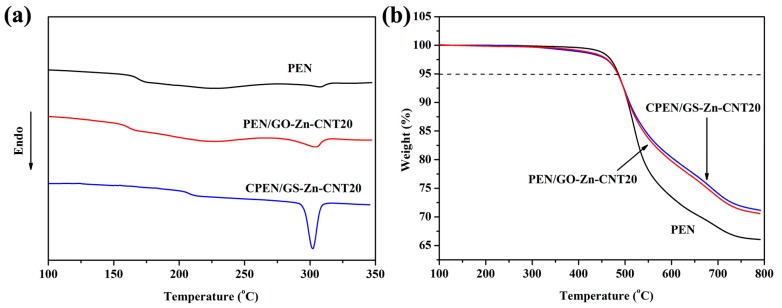
The differential scanning calorimetry (DSC) (**a**) and TGA curve (**b**) of PEN, PEN/GO-Zn-CNT20, and CPEN/GS-Zn-CNT20.

The thermal properties of the PEN and CPEN/GS-Zn-CNT based composites were investigated by DSC and TGA measurements. As shown in Figure 10a, the PEN-Ph shows a glass transition temperature (*T*_g_) at 165 °C and a melting point (*T*_m_) at 308 °C. For the composite PEN/GS-Zn-CNT20, the *T*_g_ and *T*_m_ decreased a little due to the nucleating effect of GS-Zn-CNT to the PEN matrix. However, the *T*_g_ of CPEN/GS-Zn-CNT20 was as high as 200 °C, resulting from the crosslinking of PEN which constraints the movement of the polymeric main-chain. Yang et al. reported the *T*_g_ increased by more than 100 °C for another kind of PEN after curing treatment [3]. In addition, due to the isothermal crystallization during the curing treatment at 320 °C, the melting enthalpy of CPEN/GS-Zn-CNT20 also increased. For the TGA measurement, the results show that the first decomposition temperatures (*T*_5%_) of PEN/GS-Zn-CNT and CPEN/GS-Zn-CNT were almost the same as that of PEN, which was around 500 °C (Figure 10b). Similar results were also observed for the other composites with different content of GO and CNT.

**Figure 11:**
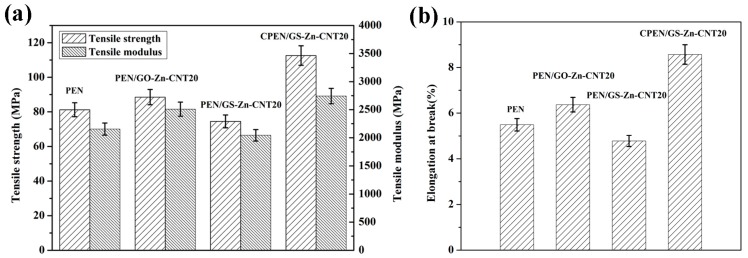
The tensile strength and tensile modulus (**a**) and elongation at break (**b**) of PEN, PEN/GO-Zn-CNT, PEN/GS-Zn-CNT20, and CPEN/GS-Zn-CNT20.

The tensile strength, tensile modulus, and elongation at break of PEN and the PEN based composites are shown in Figure 11. As a high performance polymer, PEN shows a tensile strength of 81.2 MPa and a tensile modulus of 2154.9 MPa. For the composite PEN/GO-Zn-CNT20, the tensile strength and tensile modulus increased to 88.5 and 2508.7 MPa, respectively. The increase of the mechanical properties resulted from the toughing effect and network structure of the additive GO-Zn-CNT [15]. When the composite PEN/GS-Zn-CNT20 was annealed at 230 °C for 1 h, both of the tensile strength and tensile modulus decreased due to the fact that the thermal reduction of GO generated gases which resulted in pores in the system, as shown in Figure 6c. After curing treatment at 320 °C for 2 h, the tensile strength and tensile modulus of CPEN/GS-Zn-CNT20 increasd to 112.6 and 2743.3 MPa (Figure 11a). The increment of the tensile strength and tensile modulus indicates the crosslinking of the PEN which forms the phthalocyanines as the crosslinkers in the system. The elongation at break shows a similar phenomenon as that of the tensile strength and tensile modulus (Figure 11b). For the composites with the other content of GO and CNT, similar results were also observed.

## 4. Conclusions

An organic-inorganic interpenetrating network CPEN/GS-Zn-CNT, crosslinked polyarylene ether nitrile as the organic network and the zinc ion bridged graphene sheet and carbon nanotube network as the inorganic network, was fabricated. GO and acidulated CNT were firstly prepared and then coordinated with zinc ions to form the inorganic network GO-Zn-CNT. The GO-Zn-CNT was characterized by UV-vis spectra, TGA measurement, and SEM and photograph observation. Phthalonitrile end-capped polyarylene ether nitrile permeated into the GO-Zn-CNT in NMP and the corresponding semi-interpenetrating network PEN/GO-Zn-CNT was fabricated through the solution-casting method. PEN/GS-Zn-CNT was obtained by thermal reduction of PEN/GO-Zn-CNT at 230 °C for 1 h which was confirmed by the obvious increase of the relative permittivity. By further curing at 320 °C for 2 h, which induces the self-crosslinking of PEN-Ph, CPEN/GS-Zn-CNT was finally obtained. In comparison with PEN/GS-Zn-CNT20, the relative permittivity of CPEN/GS-Zn-CNT20 decreased while the *T*_g_ of it increased due to the crosslinking of PEN. The obtained CPEN/GS-Zn-CNT showed excellent properties suggesting it can be used as material in high performance electronic devices.
